# Detecting Regulatory Mechanisms in Endocrine Time Series Measurements

**DOI:** 10.1371/journal.pone.0032985

**Published:** 2012-03-26

**Authors:** Daniel J. Vis, Johan A. Westerhuis, Huub C. J. Hoefsloot, Ferdinand Roelfsema, Margriet M. W. B. Hendriks, Age K. Smilde

**Affiliations:** 1 Department of Metabolic Diseases, University Medical Center Utrecht, Utrecht, The Netherlands; 2 Biosystems Data Analysis, Swammerdam Institute for Life Sciences, University of Amsterdam, Amsterdam, The Netherlands; 3 Netherlands Metabolomics Centre, Leiden, The Netherlands; 4 Department of Endocrinology and Metabolic Diseases, Leiden University Medical Center, Leiden, The Netherlands; The John Curtin School of Medical Research, Australia

## Abstract

The regulatory mechanisms underlying pulsatile secretion are complex, especially as it is partly controlled by other hormones and the combined action of multiple agents. Regulatory relations between hormones are not directly observable but may be deduced from time series measurements of plasma hormone concentrations. Variation in plasma hormone levels are the resultant of secretion and clearance from the circulation. A strategy is proposed to extract inhibition, activation, thresholds and circadian synchronicity from concentration data, using particular association methods. Time delayed associations between hormone concentrations and/or extracted secretion pulse profiles reveal the information on regulatory mechanisms. The above mentioned regulatory mechanisms are illustrated with simulated data. Additionally, data from a lean cohort of healthy control subjects is used to illustrate activation (ACTH and cortisol) and circadian synchronicity (ACTH and TSH) in real data. The simulation and the real data both consist of 145 equidistant samples per individual, matching a 24-hr time span with 10 minute intervals. The results of the simulation and the real data are in concordance.

## Introduction

Hormones are important agents in the regulation of physiological processes. Endocrine glands, producing hormones [1], [2], often secrete their product in short well-synchronized bursts, referred to as episodic secretion [3]. The episodic secretion depends on the circadian rhythm but also involves a strong stochastic component [4]–[6]. Secretion results in changes in hormone levels, usually leading to critical modulation of tissue function triggering the secretion of other hormones.

Hormones can thus be seen as an ensemble of initiators and inhibitors that critically modulate physiological processes. This paper refers to regulatory mechanisms in the strict context of actions between hormones, which can be initiatory, inhibitory or both. It has been shown [1], [7] that these regulatory relations between hormones are subject to the physiological state, e.g., age, gender, lifestyle and pathology, and thus exhibit some inter- and intra-individual variability. Characterizing these regulatory mechanisms can give insight in how the implementation of regulation varies among different individuals, and how such is influenced by pathology or (drug) treatment.

Ideally, characterizing the regulatory mechanisms should be based on fundamental physiological and kinetic models in which the parameters are estimated from dynamic data obtained through an optimal experimental design. Unfortunately, prior knowledge about the interactions, constants, modalities, and (inter)dependencies required for this model-based approach is often lacking and, hence, optimal experimental designs can not be defined. Common experiments are either intervention studies where the response of one hormone to another hormone is registered in an infusion experiment or time-resolved serum hormone concentrations under standard physiological conditions. The infusion experiments are laborious, invasive and do not necessarily reflect normal physiological conditions. Time-resolved concentration measurements, on the other hand, do not have these disadvantages. They also contain information about regulation albeit in a concealed form.

In this paper, we present a strategy to recover information on regulation from time series of serum hormone concentrations. More exactly, what is aimed for is to extract global information on regulatory mechanisms, such as inhibition, activation, thresholds, and circadian synchronicity. This strategy uses a set of global measures, that can summarize the relations between hormone time profiles. From these summarizing measures regulatory patterns can be inferred. The introduced measures are a set of cross-correlation profiles of the hormone time series. Considering simulated hormone times series of which the generating regulatory mechanism is known, it can be shown that different types of regulatory behavior result in different types of cross-correlation profiles. Additionally, a confirmation of the suitability of the measures is given for sets of measured hormone time series, for which regulatory mechanisms are well understood.

The information obtained on regulatory mechanisms from these measures can be exploited in several ways. From a theoretical perspective, it can be used to improve time series sampling schemes, or suggest developments in the field of fundamental mechanistic models. More importantly, from a practical perspective, it can serve as a tool to characterize variations in regulation between individuals or detect changes related to pathophysiology.

## Materials and Methods

### Endocrine time series

In observing cohorts of subjects with different features (age, sex, phenotype, lifestyle) the hormone ensemble is likely differentially regulated. Being able to characterize cohorts of subjects having a disease or lifestyle feature in common, a normality study on regulation helps to mark normal regulation and aids to distinguish it from non-normal regulation. A group of nine lean healthy volunteers, age 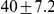 year and body mass index was 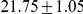 (mean 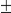 SD), consisting of four women (not pregnant and in the early follicular phase), and five men, was used in this study. The subjects were asked to refrain from strenuous physical exercise, and did not use any hormonal medication. The data are part of a normality study performed by the Department of Endocrinology of the Leiden University Medical Center, the ethical committee approved the study. All participants gave written consent. The sampling scheme comprised drawing 145 whole blood samples with 10 minute intervals over a 24-hour period. The data of this study include six hormones (adrenocorticotropic hormone (ACTH), cortisol, thyroid-stimulating hormone (TSH), luteinizing hormone (LH), follicle-stimulating hormone (FSH), and growth hormone (GH)), where quantification of hormone concentrations was performed with sensitive immunoassays. The motivation for using the lean cohort in this paper was to show the endocrine relations in healthy lean subjects. These and other statistics about the cohort are summarized in Table 1.

**Table 1:** Estradiol levels in women were obtained in the (early) follicular phase of the menstrual cycle.

*Basal characteristics of the volunteers*							
Subject	Gender	Age	BMI	Estradiol	Testosterone	IGF-1	fT4
		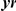	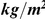	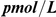	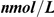	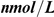	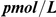
1	female	33	22.10	147	ND	18.9	15.5
2	male	36	21.60	57	15.7	23.8	21.2
3	female	33	20.59	85	ND	19.7	17.6
4	male	55	21.80	56	19.8	14.3	20.1
5	male	43	22.60	55	12.1	24.2	16.0
6	female	41	20.58	82	ND	30.6	16.5
7	male	44	22.69	54	19.1	12.3	19.6
8	female	34	20.42	40	ND	35.1	17.3
9	male	37	23.41	57	12.4	14.4	16.3

Testosterone levels in men were in the normal range (10–30 nmol/L). ND: not determined. Normal values for IGF-1 are age-dependent and range between 10–35 nmol/L. Normal free thyroxine levels (fT4) are between 10–22 nmol/L. The BMI levels are all within the non-obese range.

### Assay characteristics

Growth hormone, PRL, TSH, LH and FSH were all measured by time-resolved fluoroimmunoassays (IFMA) (Delfia, PerkinElmer-Wallac Oy, Turku, Finland). The standard (Genotropin) used in the GH assay was obtained from Pharmacia and Upjohn, Uppsala, Sweden, and calibrated against World Health Organization (WHO) Second Standard International Reference Preparation, IRP 80/505.The detection limit is 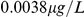. The intra-assay coefficient of variation (CV) is 1.6–8.4%. Prolactin was calibrated against the 3rd WHO standard IRP 84/500. The detection limit is 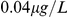 and the intra-assay CV 3–5.5%. Thyrotropin was calibrated against IRP 80/558. The detection limit is 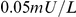 and the intra-assay CV 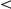
5%. LH assay is calibrated against LH standard 80/552. Detection limit is 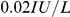, intra-assay CV 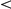
3%. FSH is calibrated against IRP 94/632. The detection limit is 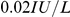, and the intra-assay CV 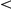
3%. ACTH was measured with an immunoradiometric assay (IRMA) (Nichols Institute Diagnostics, San Juan Capistrano, CA, USA). The detection limit is 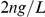, and the intra-assay CV is between 2.8–7.5%. Cortisol was measured with a radioimmunoassay (DiaSorin, Stillwater, MN, USA). The detection limit is 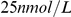, and the intra-assay CV ranges between 2–4%.

### Association measures

The minimal model that is believed to underlie endocrine time series data is composed of a secretion term and exponential decay, together explaining the variation in hormone concentration [3]:
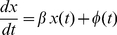
(1)Equation 1 describes the changes in time 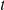 of the concentration 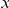, with 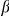 the decay constant and 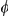 the secretion term. The identification of the parameters in such a model is not trivial as the model is ill-posed; there is a trade off between the decay constant (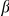) and the secretion (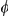). The model can successfully be parametrized by explicitly enforcing the assumption of episodic secretion through the constraint that the secretion term 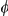 should have many zeros. Choosing the optimal number of zeros can be performed by an appropriate model selection criterion [3].

**Figure 1:**
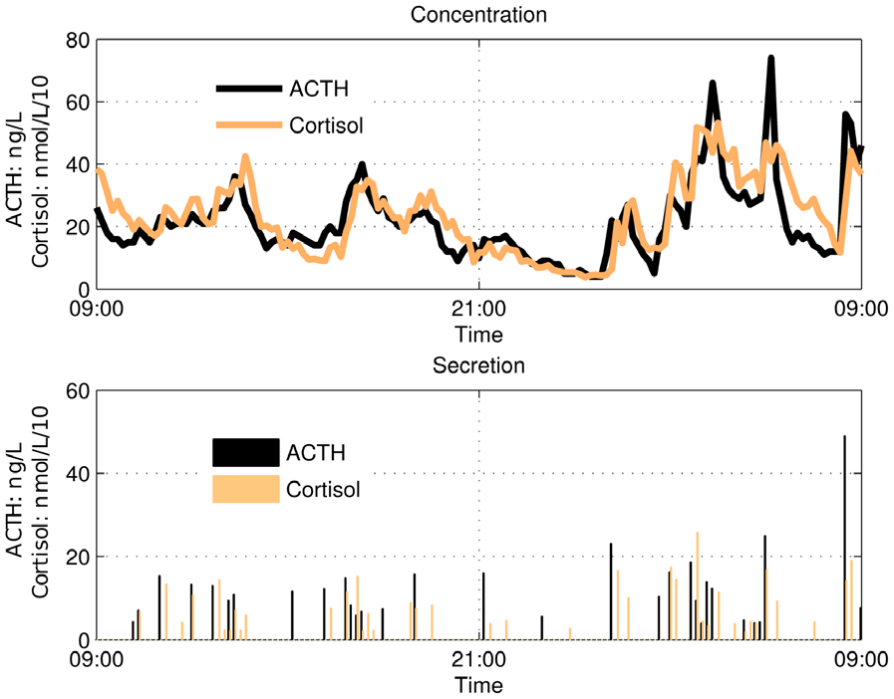
An example of measured 24-hr 10-minute interval serum hormone profiles. The dark line in the upper figure represents ACTH; the estimated secretion is shown by the dark bars in the lower panel. In this system, ACTH pulses drive or elicit pulses of cortisol pulses with a delay of approximately one time unit. Cortisol (light line, scaled by factor 0.1) also receives some pulse stimuli from auxiliary inputs as not all cortisol pulses are preceded by an ACTH pulse (see the lower figure).

The model of Equation 1 is used to extract the time series of secretion pulses (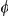) from the hormone time series, which together with the concentration level information (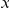) are used to construct a series of association metrics. An example of a measured concentration and an estimated secretion pulse time series is shown in Figure 1.

Four association metrics that are specific implementations of the cross-correlation function, exploit the covariation between hormones considering both concentrations and secretion pulse distribution and amplitudes. These metrics can be used to extract information on regulation between hormones. Metric AM1 describes the association between the concentration time series of two hormones. This first association metric is simply the cross-correlation function of the concentration time series of two hormones 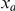 and 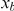 (with 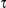 indicating the time lag, and 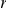 the correlation coefficient):
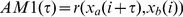
(2)AM2 describes in the same way the association between pulse time series of two hormones:
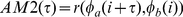
(3)Two other measures explicitly focus on the secretion induced response, by conditioning the data series on (estimated) secretion activity. One metric correlates the concentration levels of one hormone with the secretion amplitude of the second hormone conditional on the presence of secretion events of the second (AM3). The other metric correlates the concentrations of both hormones conditional on the presence of secretion events in one of them (AM4).

The conditioning takes the time points at which secretion takes place (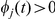) as the conditioning vector (Equation 4), being a subset of the original time vector.
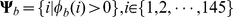
(4)where 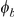 is the vector holding the secretion amplitudes of hormone *b* and 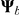 is the set of secretion indices. The AM3 metric uses the concentration of 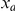 and the secretion 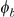 conditional on non zero secretion events in 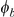. It describes the association between the concentration time series of one and the pulse time series for another hormone selecting only those time points where a pulse in this second hormone was observed.
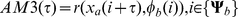
(5)The *AM3* metric, since concentrations of the first hormone are used, contains accumulated information about past events, in contrast to *AM2*, where only information on the pulse moment is considered. AM4 describes the association between the concentration time series of both hormones considering only those time indices that do pulse in one of the hormones.
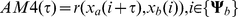
(6)Instead of pulse amplitude information, circulating hormone concentration levels are used, which might give more powerful evidence of the existence of a response in 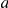 to secretion pulses of 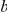. *AM4*, as compared to *AM1*, does not concentrate so much on decay patterns, but more on secretion related phenomena.

Generally, cross-correlations are mirrored around lag zero, when interchanging the labels *a* and *b* in equations 2 and 3. Since both cross-correlation profiles will yield the same information, only one of the two is depicted in the figures.

For *AM3* and *AM4* the selection of indices in Equation 4 are not identical for the two hormones involved in the cross-correlation calculation (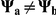). This implies that two versions of the metrics *AM3* and *AM4* exist. One where conditioning is on the secretion pulses of *a* and one for conditioning on pulses in *b*.

**Figure 2:**
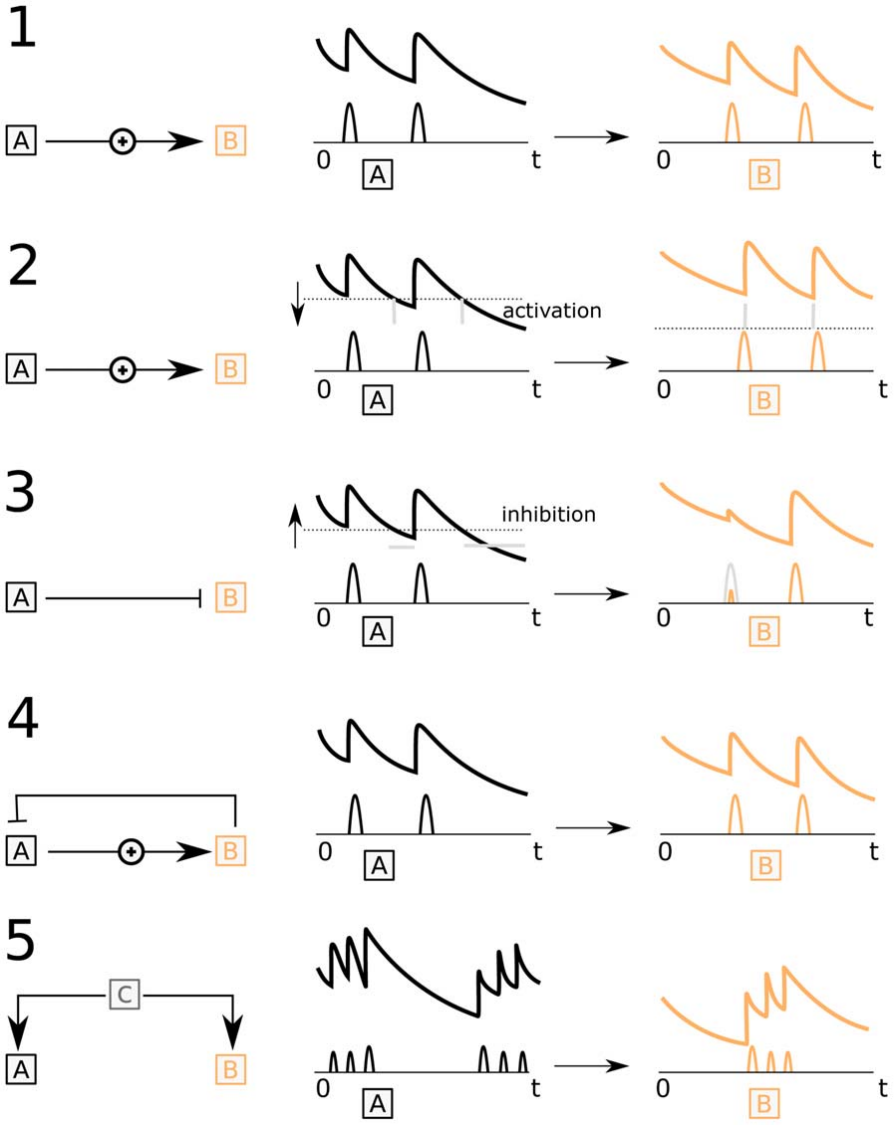
Schematic depiction of the simulated regulatory mechanisms in episodic secreting hormones. (**1**) Shows a mechanism in which a pulse in 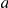 triggers a pulse in 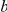. (**2**) Shows a mechanism that triggers a pulse to be released in 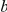 when 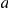 falls below a (preset) threshold. (**3**) Shows a mechanism that inhibits the pulse amplitude in 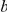 when the concentration in 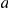 is above a (preset) threshold. (**4**) Shows that combining mechanisms (**1**) and (**2**) appear as mechanism (**1**).(**5**) Depicts a diurnal pattern that is maintained by another (shared) variable.

Inhibition is common in biology, and inhibitory actions can be found on the molecular level and on higher levels involving complex tissue responses, see Figure 2 subfigure 3. For this reason a simulation example was set up that mimicked inhibition in pulsatile systems. To this end, two sets of unrelated pulses were constructed by random sampling. The pulse series of hormone 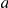 was integrated to give concentration values. Then the pulse series of hormone 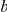 was adapted such that when the concentration in 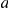 exceeded a threshold value, the pulses in 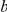 were reduced in amplitude by 90%. The resulting concentration and pulse series were then analyzed by the different association metrics.

The paper's objective is to show that regulation mechanisms can be extracted from hormone concentration data by using the proposed association metrics. To this end five different common types of regulatory mechanisms were studied, (1) rapid activation with lag (*a* activates *b*), (2) concentration threshold activation, (3) inhibition (*b* inhibits *a*), (4) a combination of inhibition and activation, (5) diurnal patterns, without direct action of one hormone on the other (see Figure 2 for a schematic representation of the regulatory mechanisms).

For each of the five types of regulatory triggering mechanisms a hundred time series were simulated and, when available, compared with real measured data (see Suppporting Information S1 for a description of the simulations). The results of the measured data series are shown with the 95% confidence interval of the mean. The confidence statistics are calculated on the Fisher-Z transformed correlation values after which the confidence interval values are transformed back to the normal correlation space. The result of this operation is that the confidence intervals are not symmetric about the mean. The case of rapid activation will be discussed and illustrated at length to show the interpretation of the metrics. It will be shown that it is possible to extract certain regulatory mechanisms from time series data.

## Results

### Rapid activation

In a stimulatory system consisting of two hormones, 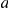 and 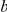, a pulse in 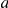 translates into a pulse in 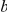. This activation system is the simplest link between two hormones as all that is required is a receptor, and a signal transduction cascade that triggers the secretion of the other hormone into the circulation, see subfigure 1 in Figure 2.

The pulses in 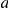 are followed by the pulses in 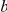 which, in this simulation study, are lagging by one sampling unit. There are no additional inputs to 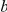 nor is there any other source of noise or (measurement) error introduced in this simulation.

**Figure 3:**
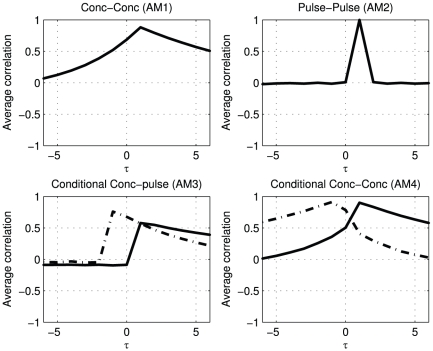
Pulses in 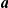 induce pulses in 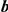 after 1 lag, without auxiliary (noise) pulses in 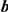. The solid black lines represent the cross correlation profiles after conditioning on 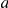, the dashed lines after conditioning on pulses in 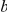.

In Figure 3 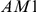 shows a clear optimum at lag 1, which matches the designed delay between pulses of the two hormones. 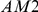, representing the cross-correlation between pulse profiles, shows clearer that there is a nonzero relation between 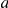 and 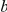 at lag 1. At other lags (by design) there is no relation, which is better represented in 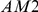 than in 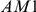. 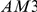 shows the relation between the pulse amplitude of one hormone at time indices with actual secretion and the concentration of a second hormone. The black solid line shows that the pulses in 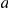 correlate strongest with the concentration of 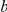 at lag 1. It is noteworthy that before lag 1 there is no correlation between the pulse amplitude and the concentration. At high lags correlations are present, which are due to the correlation of the pulse of 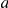 with the exponential decay profile of 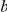. Conversely, the dashed line shows that the pulses in 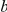 correlate strongest with the concentration in 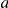 at lag −1. As is expected, on the solid line, there is a non zero relation after lag −1 and no relation before lag −1. 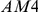 shows the relation between the concentrations at time indices with actual secretion; the cross-correlation profiles depend on the secretion of 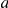 and 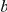. The black solid line is based on conditioning on the pulses in 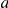 while the dashed line is produced by conditioning on the pulses in 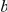. Both show a clear peak at lag 1.

**Figure 4:**
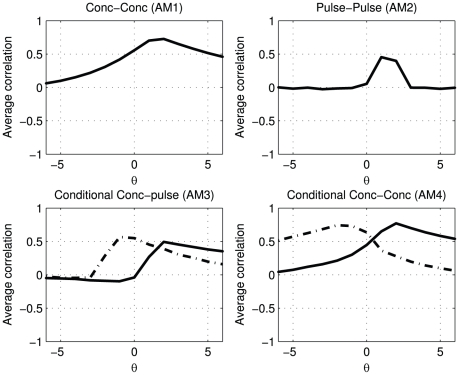
Pulses in 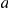 induce pulses in 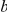 after lags of 1 or 2 (randomly drawn with an equal probability). In AM3 and AM4 the solid line is related to conditioning on pulses of 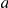 and the dashed line on the pulses of 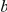.

An introduction of variation in the lag, being either 1 or 2 lags, when generating simulated data, is reflected in the association metrics (see Figure 4). Along with the previous simulation model pulse-to-pulse variation in the lag, with which a pulse in hormone 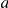 induces a pulse in hormone 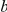, is introduced. Optimum values of the association values are now found at lag 1 as well as lag 2.

**Figure 5:**
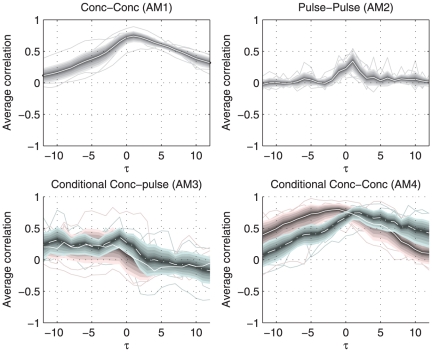
Overview of the metrics on the HPA axis hormones ACTH and cortisol. The shaded area around the white lines marks the 95% confidence interval of the mean, the thin lines show the individual results. The four metrics unanimously point to lag 1, but the different metrics center on different aspects of the relation between ACTH and cortisol.

The association measures were applied to times series measurements of adrenocorticotropic hormone (ACTH) and cortisol from 9 healthy and lean subjects. Figure 5 shows the association measures describing the relation between the two hormones. The presented cross correlation profiles are averages over individual profiles. Pulse profiles were estimated from the concentration time series using a method described in [3]. The relation between ACTH and cortisol is well known, there is rapid activation of the secretion of cortisol by ACTH. 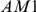 and 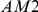 point to some optimum at lag 1, though 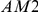 shows that the correlation at lag 0 is unequal to zero. Moreover, the true lag may be smaller than 10 minutes, e.g., between lag 0 and 1. The results are similar to those shown in Figure 4, though 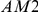 is much less pronounced. The latter is likely caused by subject to subject variation in the optima of 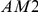. The 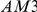 results are different from those of the simulation study. It is postulated here that this difference is related to temporal clustering of pulses and the circadian rhythm that are not included in the simulation model. The slight differences observed between 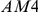 and the simulation model need to be interpreted in the same terms.

### Threshold activation

**Figure 6:**
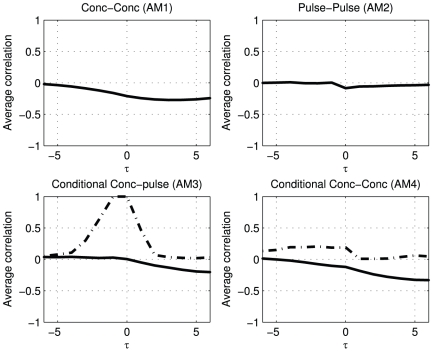
A concentration drop of 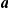 below a certain threshold induces a pulse in 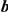. In AM3 and AM4 the solid line marks the conditioning on pulses of 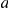 and the dashed line the pulses of 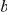.

An activating system is considered which creates a single trigger secretion pulse in 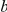 when the concentration of 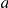 falls below a relative set point, see Figure 2 subfigure 2. This is a conditional activating system that yields strikingly different results, as shown in Figure 6, when compared to the rapid activation system. The 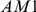 and 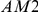 results are weakly negative from lag 0 and up, as expected, since low concentrations of hormone 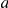, will induce pulses in 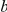 and therefore show negative correlation patterns. The 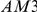 elucidates a striking pattern in the relation between pulses of 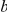 and the concentrations of 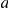. The conditioning on pulses of 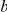 correlates these pulses with the decaying pattern of hormone 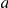 before it drops below the threshold, when it induces a pulse in 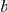. The patterns for the four association metrics are very different for rapid activation and threshold activation, serving the goal of this study.

### Inhibition

**Figure 7:**
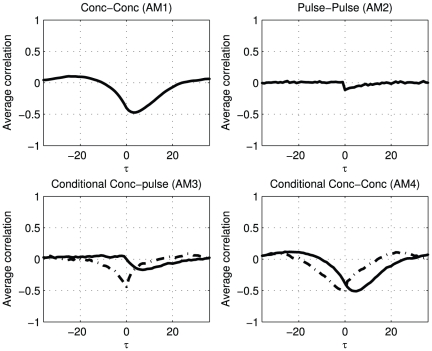
The association metrics for a two hormone system in which increased concentrations of 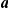 diminish the pulse amplitudes of 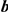 by 90%.

Figure 7 shows that inhibition can be identified by negative associations, especially for the concentration based measures 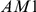 and 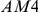. When the concentration in 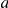 is high it will diminish the secretion of 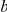. The small values for the associations as measured with 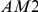 are caused by the fact that the pulses of 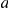 and 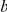 are generated randomly and independently. The negative associations found for inhibition contrast with the positive associations found for rapid activation, and with the very distinguishing patterns for concentration threshold activation.

### Activation and inhibition

**Figure 8:**
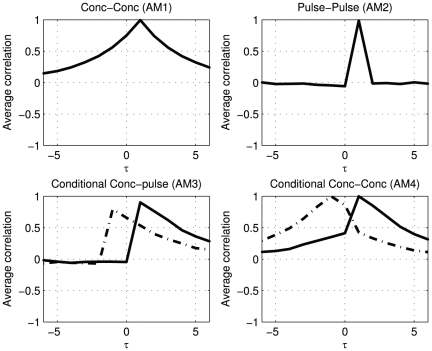
The association metrics in a system in which 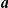 drives 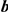 with a time lag of one and 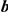 acts as an inhibitor when the concentration of 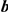 exceeds a certain threshold such that the new pulses in 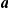 are reduced by 90%.

Many biological systems are thought of as being regulated by a combination of activation and inhibition, see Figure 2 subfigure 4. The tight integration of the two mechanisms especially allows for a finely regulated system. This poses some intriguing questions for the analysis as variation is believed to be dominated by secretion. When the negative inhibition becomes active, the new secretion episodes are dampened, meaning that active inhibition cannot be detected directly but needs to be deduced from the absence (or diminishing amplitudes) of secretion pulses in the inhibition target. Mechanistically, this system can be a self limiting system composed of two components. Some formalized argumentation is provided in the Supporting Information S1 (see Equations S.13, S.14 and S.15). The result of the association metrics of a set of hormones in a system with strong inhibition is shown in Figure 8. There are no differences between Figure 8 and 3, indicating that the two systems can not be distinguished from each other based on the proposed metrics. The examination of the distribution of pulse amplitudes of the inhibition target conditional on the (lagged) concentrations of the inhibitor may reveal inhibition. However, for real data it is questionable if this kind of information can be retrieved as data on the inhibited as well as the uninhibited pulse amplitude distributions need to be available.

### Diurnal patterns

**Figure 9:**
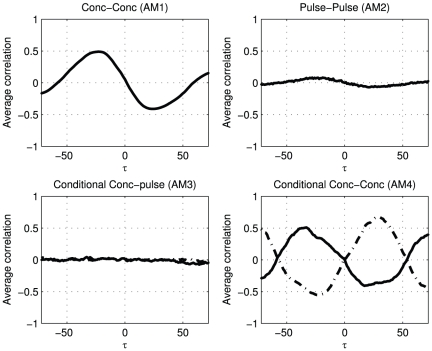
The association metrics of simulated concentration profiles from a simple system of two hormones exhibiting diurnal patterns, but without direct activation or inhibition.

Many hormones are secreted in diurnal patterns. The processes that drive the secretion of such hormones are not required to be causally dependent on each other, see Figure 2 subfigure 5. No physiological relation has to exist between the secretion pulses other than the cyclical temporal association of the secretion processes. The observed association patterns (Figure 9) are the result of diurnal behavior that presents itself with similar fluctuation patterns, apart from some time shift. This type of associations shows a particular wave form and is most dominant for the concentration profiles (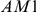 and 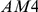).

**Figure 10:**
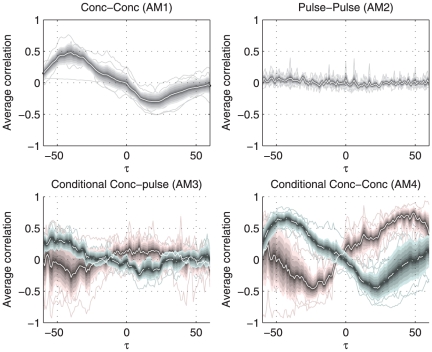
Overview of association metrics of ACTH and TSH. The shaded area around the white lines marks the 95% confidence interval of the mean, the thin lines show the individual results. The metrics point at a relation at two optima at lags −42 and 22.

An example of this type of association in real data is given in Figure 10. The associations of ACTH and thyroid-stimulating hormone (TSH) extracted from hormone concentration profiles of 9 lean controls, the AM1 and AM4 plots reveal similar patterns with two optima. ACTH and TSH are not believed to directly regulating each other. A potential modulator that regulates the action of both hormones, can possibly found outside this two-hormone system in corticotropin-releasing hormone (CRH) and thyrotropin-releasing hormone (TRH) levels.

## Discussion

The identification of pulse patterns in hormone concentration time series, combined with the estimation of association measures can reveal the functional relations between hormone pairs. Basic biological regulatory mechanisms were investigated, and association measures were evaluated on their ability to distinguish these mechanisms. In a rapid activation system such as presented in two simulation studies, the 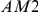 clearly unmasks the underlying mechanism and is the best metric for this type of mechanism. This was also confirmed by the real system of ACTH-cortisol measurements. On the whole the mechanisms show distinct association measure patterns, making it possible to determine regulatory mechanisms based on hormone concentration profiles. The exception is when activation is combined with inhibition. Without additional information about the activation process, and the secretion of the activating hormone, no information on the inhibition can be extracted. Diurnal behavior of hormones also shows distinct association patterns. This may give rise to the hypothesis of the existence of a regulatory relation, however, the associations are calculated within the ‘closed system’ assumption which cannot exclude influences from outside the ‘system’. The detected diurnal relations are, in a broader context, the result of regulatory relations.

In the proposed explorative approach for revealing functional relations, basal secretion is not included in the simulations, though there are good indications that at least some hormones have a basal secretion [1], [7]. The undesired increase in the simulation complexity is the reason for not including basal secretion. Another motivation for not including basal secretion is that it can be hard to identify without the (extensive) use of prior information.

The generic detection of relations in time series with episodic activity was our motivation for developing an assumption-free set of metrics to detect regulation and diurnal relations, which is what we describe in this manuscript. An alternative strategy to using association measures for the inference of (functional) relations would be an approach where a pharmacological model, expressed in differential equations, is used. Keenan and Veldhuis *et al* [8], [9] show that based on this approach dose-response curves can explicitly be estimated based on time series hormone data. Keenan and Veldhuis *et al* [8], [9] showed that such an approach is tractable and can provide valuable information about the differences between, for instance, age groups. Our strategy, as described in the method section, on the contrary, uses very few assumptions and focuses on detecting relations, opposed to fitting a complex set of equations to data.

In this study it is shown that regulatory mechanisms can be detected in time series data. In hypothesis generation it is very valuable to probe observed concentration profiles with the association metrics that are proposed here. This approach can be used to compare regulatory behavior between cohorts. Changes in cross correlation profiles may point to changes in regulatory behavior due to disease or treatment. This will be the subject of future study.

## Supporting Information

Supporting Information S1 The supplementary material describes the mathematical details of the simulations.
(PDF)
