# Supplementary material for: Detecting Regulatory Mechanisms in Endocrine Time Series Measurements
Source: PLoS One. 2012 Mar 26;7(3):e32985. doi: 10.1371/journal.pone.0032985 (PMC3312884; doi:10.1371/journal.pone.0032985)
Supplement: Supporting Information S1 — The supplementary material describes the mathematical details of the simulations. (PDF) [file pone.0032985.s001.pdf]

S1 Supplementary material

The mathematical details of the association metrics and the simulation of concentration time series with episodic secretion are described.

S1.1 Model

Let x_i be a concentration time series in which i represents the hormone. In the following sections we will use $i = a$ or $i = b$ to designate the two hormones. Let $\phi_i(t)$ be the secretion amplitude at time t. The concentration $x_i(t)$ at a given time is determined by the previous concentration ($x_i(t-1)$), the exponential decay (β_i) and the secretion at that time ($\phi_i(t)$). A method for estimating β_i and $\phi_i(t)$ from measured series is described in Vis *et al* [1]. Hormone concentration time series are generated by:

$$x_i(t) = \beta_i x_i(t-1) + \phi_i(t) \tag{1}$$

In the simulation studies, for all hormones, the decay parameter β_i is set such that the half life is 5.5 time units; assuming that the time units are separated by 10 minutes this translates to a half life of 55 minutes. A shorter half life reduces the carry-over of events, when taken to the extreme, the concentration and secretion values will converge. A different half life value will affect $AM1$, $AM3$ and $AM4$, but not $AM2$.

S1.2 Calculation of metrics for real measured and simulated data

All presented association metrics are averaged cross-correlation profiles of 100 randomly generated datasets. The model mechanisms are simulated by independently generating 100 time series of two hormones, each at 145 time indices, the averaged result is presented in the figures. For the real data in Figures 5 and 10 the average profiles are based on data for 9 healthy subjects. The presented association measures are averaged over the 9 individual association measures.

The association metrics proposed are based on Pearson correlation which has a skewed sampling distribution. This skewing is lessened by taking the Fisher Z transform of the correlation which means taking the arc tangent hyperbolic [2,3]. The reported values are Fisher Z values that are transformed back to the original domain by taking the tangent hyperbolic. The results of the measured data are reported with the 95% confidence bounds of the mean. These values are calculated on the Fisher Z transformed data and then transformed back to the normal correlation space.

S1.3 Simulation of time series

S1.3.1 Rapid activation

The activation mechanism dictates that a secretion pulse in one hormone will give a pulse in the second hormone, but with some delay. The following equations show how the time series data were generated:

$$\begin{aligned}
\eta_a(t) &\sim B(1,p),\ p \in [0,1] && (2)\\
\Phi_a(t) &\sim \chi_1^2 && (3)\\
\phi_a(t) &= \eta_a(t)\Phi_a(t) && (4)\\
\phi_b(t+1) &= \alpha\eta_a(t)\Phi_a(t) && (5)
\end{aligned}$$

The $\eta_a(t)$ defines whether secretion occurs in the first hormone (a) at time t based on a Bernoulli distribution with success probability p, and $\mathbf{\Phi}_a(t)$ defines the amplitude of such a pulse. Each element

of the pulse vector $\eta_a(t)$ is drawn from a binomial distribution with probability p. The realized secretion $\phi_a(t)$ activates secretion of the other hormone (b) described by $\phi_b(t+1)$, where α is a positive scalar.

The delay of translating a pulse in a to the secretion of a pulse in b may vary. Equation 6 defines such variability resulting in Equation 7 in which ζ denotes the variable delay. As a result, with an equal probability, the pulse translation takes a delay of 1 or 2 time units. A tilde is added to $\tilde{\phi}_b$ to distinguish it from ϕ_b shown in Equation 5.

$$\zeta \sim B(1, 0.5) \tag{6}$$

$$\tilde{\phi}_b(t+1+\zeta) = \alpha\eta_a(t)\Phi_a(t), \tag{7}$$

S1.3.2 Threshold activation

Concentration profiles of hormone a are generated using Equations 2 to 4 followed by Equation 1. A concentration threshold as a pulse triggering mechanism, is implemented such that when the concentration of a falls below a preset threshold (see Equation 8) this induces a single pulse in b.

$$\flat(t,\vartheta) = \begin{cases} 1 & \text{if } x_a(t) < \vartheta \wedge x_a(t-1) \geq \vartheta \\ 0 & \text{otherwise} \end{cases} \tag{8}$$

$$\check{\tilde{\phi}}_b(t+1) = \alpha\eta_a(t)\flat(t,\vartheta)\Phi_a(t) \tag{9}$$

S1.3.3 Inhibition

For hormone a pulse and concentration profiles are generated using Equations 2 to 4 followed by Equation 1. Based on the concentration profiles inhibition of secretion of hormone b takes place. Equation 10 calculates inhibition time points. Equations 2 and 3 are used to generate pulse instances and amplitudes for hormone b. Equation 11 shows how an inhibition of secretion of hormone b by hormone a is effectuated. A breve is added to ϕ_b to distinguish it from ϕ_b shown in Equation 5

$$\flat(t,\vartheta) = \begin{cases} 1 & \text{if } x_a(t) > \vartheta \\ 0 & \text{otherwise} \end{cases} \tag{10}$$

$$\breve{\phi}_b(t) = \eta_b(t)\Phi_b(t) - \frac{9}{10}\flat(t,\vartheta)\eta_b(t)\Phi_b(t) \tag{11}$$

S1.3.4 Activation and inhibition

For the sake of argument, the assumption is that a activates b very quickly ($\tau = 0$), the responses in b being proportional (α) to those in a. Additionally, assume that the inhibition is very quick as well. For hormone a pulse and concentration profiles are generated using Equations 2 to 4 followed by Equation 1. Now Equation 2 and 3 are used to generate pulse indices and amplitudes for hormone b. Now Equation 12 describes the pulses induced in b, since the activation is very fast, the secretion of this hormone can already be seen at the same sampling time (that is, no delay).

$$\phi_b(t) = \alpha\eta_a(t)\Phi_a(t) \tag{12}$$

The inhibition of the secretion of hormone a by hormone b can now be simulated using the following equations, $\breve{\phi}_a$ are the secretion values in the inhibited case:

$$\flat(t,\vartheta) = \begin{cases} 1 & \text{if } x_b(t) > \vartheta \\ 0 & \text{otherwise} \end{cases} \tag{13}$$

$$\breve{\phi}_a(t) = \eta_a(t)\Phi_a(t) - \frac{9}{10}\flat(t,\vartheta)\eta_a(t)\Phi_a(t) \tag{14}$$

Equation 4 defines how the secretion vector is defined from the incidence vector and the amplitude vector. Equation 14 shows an example of inhibition that limits the amplitude by 90% and is active only when Equation 8 is 1 (true).

Inhibition results in lower than normal secretion amplitudes implying that the inhibited vector will be with lower values compared to the uninhibited amplitude vector. Equation 15 shows that behavior in symbolic form, stating that the average of the inhibited amplitudes is always lower than or equal to that of the uninhibited vector. In this simulation study the uninhibited vector is known and the previous statement can be corroborated, however, when the vector is estimated from real data only the inhibited vector is known.

$$\frac{1}{T}\sum_{t=1}^{T}\breve{\phi}_a(t) \leq \frac{1}{T}\sum_{t=1}^{T}\phi_a(t) \tag{15}$$

However, the AM2 results for both sets of vectors are identical.

$$r(\phi_{\mathbf{a}}, \phi_{\mathbf{b}}) = r(\breve{\phi}_{\mathbf{a}}, \breve{\phi}_{\mathbf{b}}) \tag{16}$$

It is therefore concluded that in a system specified by Equations 13 and 11 it is impossible to assess whether $\vartheta < \infty$ which means that inhibition cannot be identified in such an approach.

S1.3.5 Diurnal patterns

To illustrate the association patterns for two unrelated hormones, concentration time series are simulated using two unrelated and different event distributions over the day. The length of the vector of events is 145. The binomial probabilities with which η_a is generated are $\frac{20}{40}, \frac{5}{65}, \frac{25}{40}$ for indices $[1 \cdot 40], [41 \cdot 105], [106 \cdot 145]$. The binomial probabilities for η_b are $\frac{10}{20}, \frac{5}{55}, \frac{30}{50}, \frac{3}{20}$ for indices $[1 \cdot 20], [21 \cdot 75], [76 \cdot 125], [126 \cdot 145]$.

$$\Phi_a(t) \sim \chi_1^2 \tag{17}$$

$$\Phi_b(t) \sim \chi_1^2 \tag{18}$$

$$\phi_a(t) = \eta_a(t)\,\Phi_a(t) \tag{19}$$

$$\phi_b(t) = \eta_b(t)\,\Phi_b(t) \tag{20}$$

This creates two distinct, but unrelated, diurnal patterns.

References

1. Vis DJ, Westerhuis JA, Hoefsloot HCJ, Pijl H, Roelfsema F, et al. (2010) Endocrine pulse identification using penalized methods and a minimum set of assumptions. Am J Physiol Endocrinol Metab 298: E146-E155.

2. Bond C, Richardson K (2004) Seeing the fisher z-transformation. Psychometrika 69: 291-303.

3. Fisher RA (1935) The Design of Experiments. UK: Edinburgh: Oliver and Boyd.
